# Shorter X-ray refractive lens design: a large-aperture single-lens dual-focus kinoform lens based on an oval curved surface

**DOI:** 10.1107/S1600577526007551

**Published:** 2026-08-25

**Authors:** Yaowu Zhang, Yuanze Xu, Tianchong Zhang, Bo Wang, Longlong Wu, Futing Yi, He Lin, Jing Liu

**Affiliations:** a Institute of High Energy Physics Chinese Academy of Sciences Beijing100049 People’s Republic of China; b University of Chinese Academy of Sciences Beijing100049 People’s Republic of China; c Shanghai Synchrotron Radiation Facility, Shanghai Advanced Research Institute Chinese Academy of Sciences Shanghai201210 People’s Republic of China; d Quanzhou University of Information Engineering, Fujian362008, People’s Republic of China; RIKEN SPring-8 Center, Japan

**Keywords:** X-rays, refraction focusing lenses, oval-kinoform lens, beam propagation method

## Abstract

At synchrotron radiation facilities, X-ray focusing elements play a crucial role. To solve the absorption problem caused by X-rays through the lens, Aristov and co-workers [Aristov, Grigoriev, Kuznetsov, Shabelnikov, Yunkin, Weitkamp, Rau, Snigireva, Snigirev, Hoffmann & Voges (2000), *Appl. Phys. Lett.***77**, 4058–4060] proposed the structure of the kinoform lens and fabricated it, greatly reducing the absorption of X-rays by the lens. However, the absorption of the kinoform lens remains substantial and the lens is very long. To address these issues, this paper proposes a novel kinoform lens optimized through an oval surface profile for its stepped structure and evaluates the lens performance using the beam propagation method.

## Abstract 2

This paper presents a novel kinoform refractive lens and utilizes the beam propagation method to simulate the focusing performance of the lens. The results demonstrate that this lens not only enhances the gain and reduces the FWHM but also reduces the lens length.

## 1. Introduction

With the emergence of fourth-generation synchrotron radiation facilities (Shin, 2021 ▸) as a research hot spot, X-ray focusing elements have gradually garnered significant attention from scientists. X-ray nanoprobe technology (Giannini *et al.*, 2020 ▸; He *et al.*, 2024 ▸; Ice *et al.*, 2011 ▸; Mino *et al.*, 2018 ▸), as a powerful tool for characterizing nanoscale samples, has placed increasingly stringent demands on X-ray focusing elements. The gain at the focal point and the size of the focal spot are critical performance metrics for X-ray focusing elements, as they determine the spatial resolution of nanoscale probe microanalysis techniques. Therefore, optimizing X-ray focusing elements aims to achieve higher gain and a smaller focal spot.

X-ray focusing elements can be broadly categorized into three types: reflective (Kitamura *et al.*, 2022 ▸; Lider, 2022 ▸; Liu *et al.*, 2012 ▸), diffractive (Chao *et al.*, 2012 ▸; Chapman *et al.*, 2021 ▸; Tong *et al.*, 2023 ▸) and refractive (Karvinen *et al.*, 2014 ▸; Kohn, 2022 ▸; Snigirev *et al.*, 1996 ▸). Among these, refractive optical elements possess inherent advantages in terms of stability and adjustability compared with the other two types, making them widely utilized, particularly in the precise control of fine beams and long focal lengths in low-emittance synchrotron radiation and free-electron laser applications. Since the initial proposal in 1996 (Snigirev *et al.*, 1996 ▸), the surface profile of compound refractive lenses (CRLs) has undergone multiple optimizations to reduce spherical aberrations. These optimizations include circular profiles with identical apertures in 1996, parabolic profiles with identical apertures in 1999 (Lengeler *et al.*, 1999 ▸), and parabolic profiles with varying apertures (AFL) (Patommel *et al.*, 2017 ▸) and oval profiles with varying apertures in 2017 (Sutter & Alianelli, 2017 ▸). During this period, to reduce absorption, a lens with a stepped structure based on diffraction theory was proposed in 2000 and named the kinoform lens (Aristov *et al.*, 2000 ▸; Liao *et al.*, 2016 ▸), and it has demonstrated further enhanced focusing performance compared with CRLs.

Sutter and Aliannelli first derived the solution to the X-ray refocusing problem and implemented the obtained surface in the surface profile design of X-ray focusing lenses (Sutter & Alianelli, 2017 ▸; Sutter & Alianelli, 2019 ▸). Xu and co-workers then proposed a novel aperture iterative formula, which was incorporated into the design of oval lenses (Xu *et al.*, 2022 ▸). Utilizing LAGA lithography technology, the fabrication of these lenses was accomplished. According to knife-edge measurements, the lens can focus X-rays to a width of 70 nm (Lu *et al.*, 2025 ▸; Xu *et al.*, 2026 ▸). While the lens can effectively enhance focusing performance, it still suffers from significant absorption and excessive length. To address these issues, this paper proposes a novel oval-kinoform lens, referred to as the OK3 lens. A comparative analysis is conducted between the proposed lens and conventional oval lenses to give a quantitative evaluation of their focusing characteristics.

To investigate the focusing performance of the novel lens, the beam propagation method (BPM) (Van Roey *et al.*, 1981 ▸) was employed for simulation. This method efficiently solves the Helmholtz equation by utilizing the split-step operator approach. During the process of calculation, an approximation is made for the refractive indices 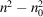 and 2*n*_0_(*n* − *n*_0_). Consequently, this method is widely applied in X-ray optics simulations (Chen *et al.*, 1998 ▸), as well as for the propagation of light at other wavelengths through a homogeneous medium. However, due to this approximation, it is not applicable to the optical simulation of visible light at interfaces. For X-ray kinoform lenses, the BPM cannot be used to give an accurate simulation of scenarios involving a high numerical aperture and the presence of back-reflection. Nevertheless, this method rigorously accounts for refraction, diffraction and absorption of X-rays at different interfaces. In this study, the BPM was applied for all lens simulations.

**Figure 1:**
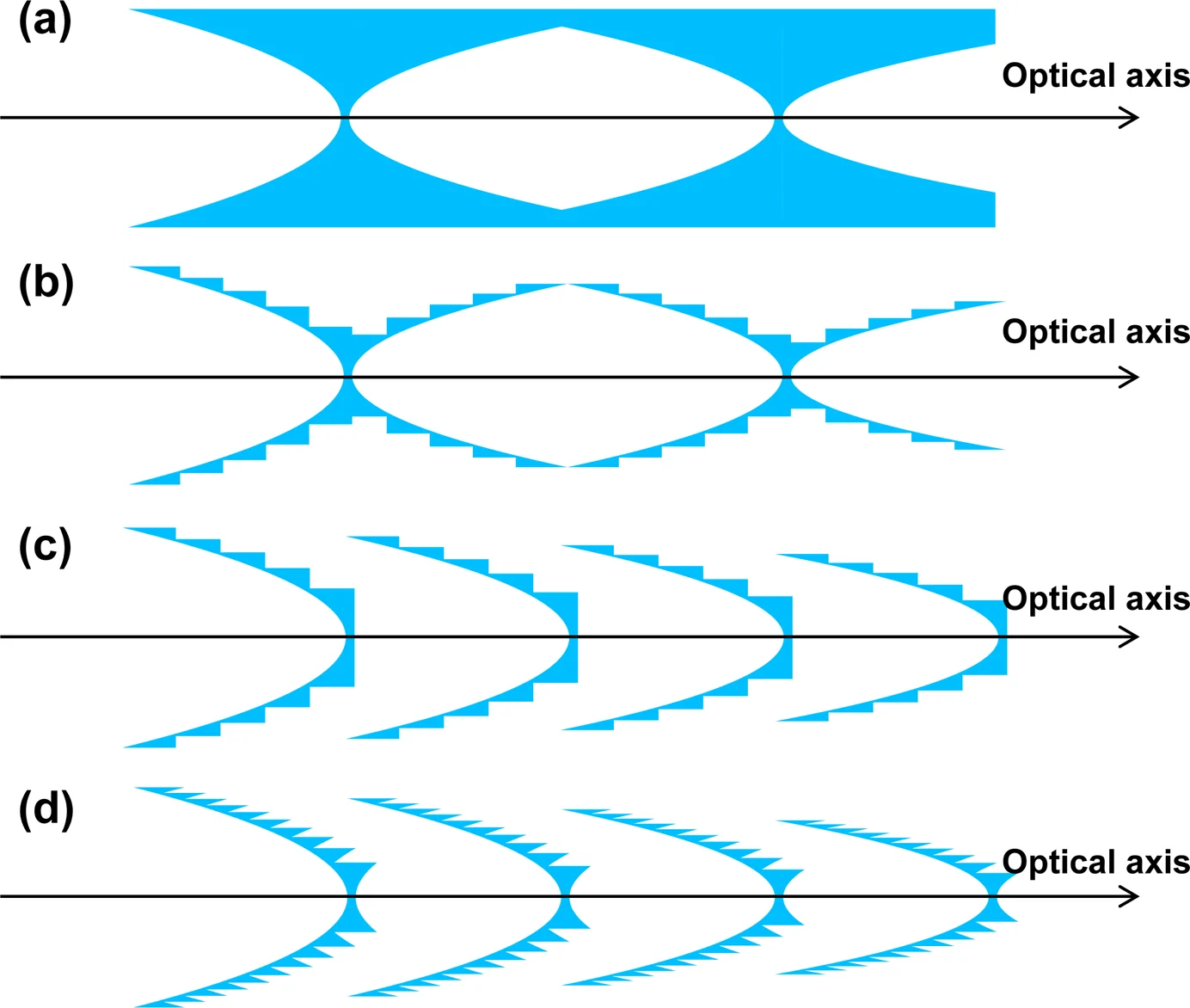
Schematic diagrams of four different lens designs. (*a*) OC lens with alternating aperture orientations, (*b*) OK1 lens with alternating aperture orientations, (*c*) OK2 lens with uniform aperture orientations and (*d*) proposed OK3 lens with optimized phase compensation.

This paper initially introduces a novel kinoform lens (OK3) designed on an oval surface, as depicted in Fig. 1 ▸(*d*). The focusing performance of this lens is then investigated through simulations using the BPM, and a comparative analysis is conducted with other oval lenses. The OC (oval-CRL) [the varying-aperture CRL lens with the oval profile depicted in Fig. 1 ▸(*a*)], OK1 [the varying-aperture kinoform lens with the same oval profile depicted in Fig. 1 ▸(*b*)], OK2 [the varying-aperture kinoform lens with the same oval profile and identical opening orientation depicted in Fig. 1 ▸(*c*)] and OK3 all utilize the configuration of variable apertures. Finally, a comparative analysis was conducted specifically between OK2 and OK3, both having the same nesting length.

## 2. Design of the OK3 lens

While the Cartesian oval curve solved by Sutter & Alianelli (2019 ▸) provides a point-to-point refocusing capability, its inherent formulation cannot accommodate collimated X-rays, thus necessitating independent optimization of the initial interface. Within the optical design framework of this work, X-rays propagate in a left-to-right configuration, with the refractive index defined as *n*_v_ = 1 in a vacuum and *n*_m_ = 1 − δ + *i*β in the lens medium. The working length is geometrically defined as the longitudinal distance between the vertex of the refractive surface (origin of the local coordinate system) and the focal point. The following sections present key parameters of the OK3 lens, including the initial surface profile function, the exit surface profile function of each kinoform lens and the aperture transfer function between adjacent lenses.

### 2.1. The initial surface profile

**Figure 2:**
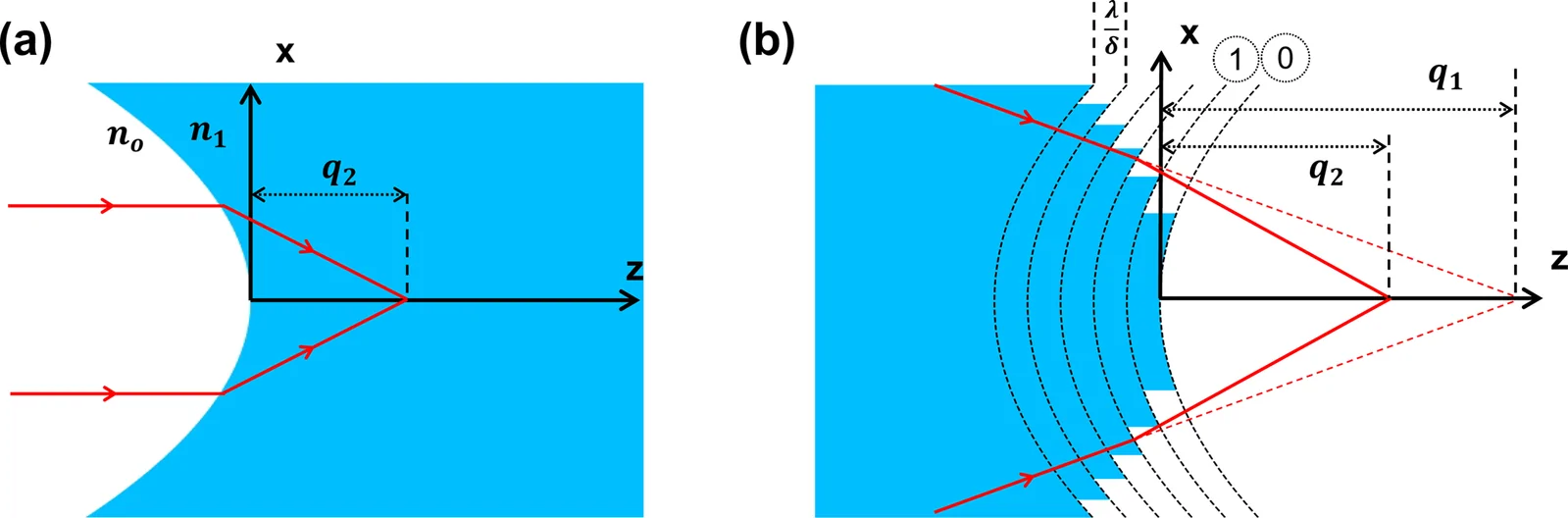
Schematic diagrams of individual surface profiles of the OK3 lens. (*a*) Initial surface profile. The left-hand side (vacuum) has a refractive index of *n*_0_ and the right-hand side (material) has a refractive index of *n*_1_. This curved surface provides an X-ray refractive focal length *q*_2_. (*b*) Exit surface profile of the kinoform lens. The left-hand side (material) has a refractive index of *n*_0_ and the right-hand side (vacuum) has a refractive index of *n*_1_. The incident focal length *q*_1_ is transformed to *q*_2_ after refraction through this surface, with a step height of λ/δ.

As illustrated in Fig. 2 ▸(*a*), when parallel X-rays transition from a vacuum into a medium through an interface, Fermat’s principle dictates that the interface function must satisfy *n*_1_[(*q*_2_ − *z*)^2^ + *x*^2^]^1/2^ = −*n*_0_*z* + *n*_1_*q*_2_ to ensure the convergence of all beams at a single focal point. Here, the vacuum refractive index is defined as *n*_0_ = 1, while the medium exhibits a complex refractive index *n*_1_ = 1 − δ + *i*β, with a specified focal length of *q*_2_.

Through calculation, the relationship between *z* and *x* can be expressed as
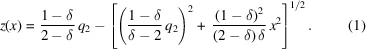


### 2.2. The exit surface profile of each kinoform lens

Building upon the Cartesian oval framework established by Sutter & Alianelli (2019 ▸), this novel lens design achieves substantial optimization over conventional kinoform lenses; their theoretical framework demonstrates that achieving a transformation in X-ray beam focusing from a working length of *q*_1_ to *q*_2_ is governed by the interface function
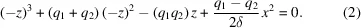
The relative refractive index decrement is defined as 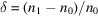, where *n*_0_ denotes the refractive index of the initial medium before refraction and *n*_1_ represents the refractive index of the subsequent medium after refraction. Therefore, in the proposed lens design [Fig. 2 ▸(*b*)], the refractive indices are defined as *n*_0_ = *n*_m_ and *n*_1_ = *n*_v_.

The exit surface profile of each kinoform lens exhibits enhanced X-ray focusing capabilities compared with conventional designs. The focal lengths before and after refraction are denoted *q*_1_ and *q*_2_, respectively. In the stepped structure inherent to kinoform lenses, the curved surfaces of individual terraces are numbered sequentially from right to left. As demonstrated by Xu *et al.* (2022 ▸), minimal absorption and optimal focusing occur when the step height equals 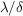. Consequently, the adjusted focal lengths for the *m*th terrace are governed by
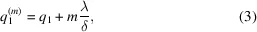

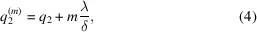
where *m* indexes the terrace position within the cascaded structure.

### 2.3. Aperture transfer function between adjacent lenses

**Figure 3:**
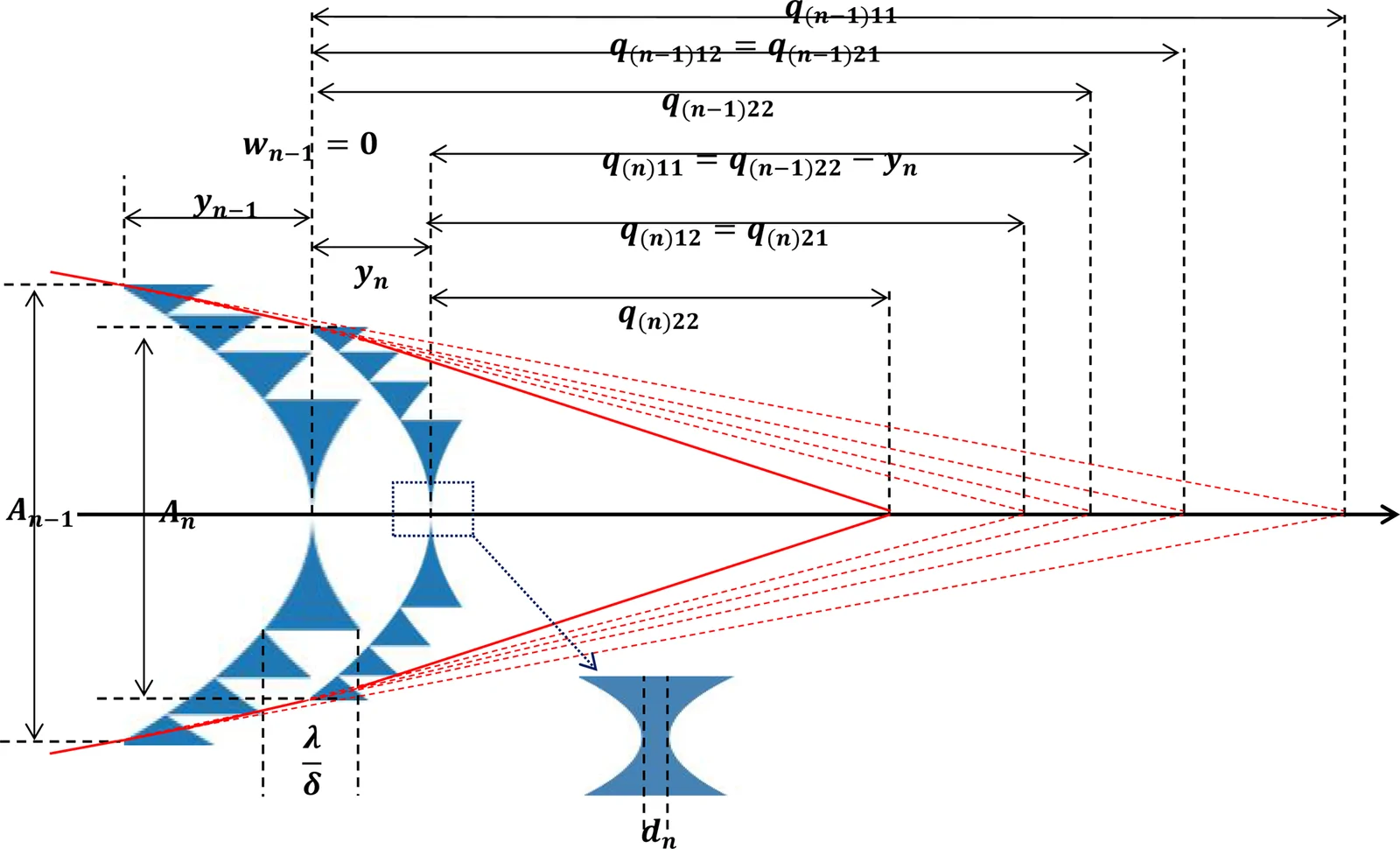
Schematic diagram of the OK3 lens focusing mechanism, showing the collaborative focusing behavior of the (*n* − 1)th and *n*th kinoform lenses in the cascaded system, where *n* = 2, 3, 4,…. Key parameters include *y*_*n*_ (axial length of the *n*th lens), *A*_*n*_ (aperture diameter of the *n*th lens), *w*_*n*−1_ [overlap length between the (*n* − 1)th lens and the *n*th lens; in this figure, *w*_*n*−1_ = 0], *d*_*n*_ (thickness of the *n*th kinoform lens), *q*_*n*11_ (pre-focusing focal length at the left-hand surface of the *n*th lens), *q*_*n*12_ (post-focusing focal length at the left-hand surface of the *n*th lens), *q*_*n*21_ (pre-focusing focal length at the right-hand surface of the *n*th lens) and *q*_*n*22_ (post-focusing focal length at the right-hand surface of the *n*th lens), with subscript indices systematically denoting lens position and optical state.

The X-ray compound refractive lens (CRL) is a multi-element optical system employing cascaded refractive lenses to achieve enhanced focusing performance through a variable-aperture configuration. As shown in Fig. 3 ▸, the X-ray beam undergoes four-stage focusing through two sequentially arranged kinoform lenses labeled *n* − 1 and *n* (where *n* = 2, 3, 4,…), denoting the lens position in the left-to-right assembly order. Key geometric and optical parameters are defined as follows:

(i) *y*_*n*−1_, *y*_*n*_: axial lengths of the (*n* − 1)th and *n*th lenses, respectively;

(ii) *A*_*n*−1_, *A*_*n*_: aperture diameters of the (*n* − 1)th and *n*th lenses, respectively;

(iii) *q*_(*n*−1)11_, *q*_(*n*)11_: pre-focusing focal lengths at the left-hand surfaces of the (*n* − 1)th and *n*th lenses, respectively;

(iv) *q*_(*n*−1)12_, *q*_(*n*)12_: post-focusing focal lengths at the left-hand surfaces of the (*n* − 1)th and *n*th lenses, respectively;

(v) *q*_(*n*−1)21_, *q*_(*n*)21_: pre-focusing focal lengths at the right-hand surfaces of the (*n* − 1)th and *n*th lenses, respectively;

(vi) *q*_(*n*−1)22_, *q*_(*n*)22_: post-focusing focal lengths at the right-hand surfaces of the (*n* − 1)th and *n*th lenses, respectively.

Under ideal conditions, the kinoform lens exhibits zero thickness (*d*_*n*_ = 0), resulting in a transfer function relationship between successive focal lengths *q*, expressed as
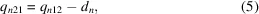

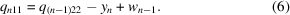
In this framework, *d*_*n*_ denotes the thickness of the *n*th kinoform lens and *w*_*n*−1_ represents the overlap length between the (*n* − 1)th lens and the *n*th lens, which is constrained as a constant throughout the theoretical analysis.

A nano-refractive focusing lens with variable apertures exhibits superior focusing performance compared with the uniform-aperture lens. To enhance the focusing capabilities further, the proposed lens design incorporates an optimized aperture modulation profile. Neglecting aperture reduction induced by X-ray propagation within the medium, the aperture transfer function can be derived through geometric similarity principles as
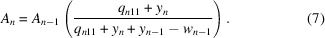
In this derived expression, *A*_*n*−1_ corresponds to the aperture diameter of the preceding lens element [the (*n* − 1)th lens] in the cascaded optical system.

## 3. Simulation and results by BPM

As can be seen from Fig. 1 ▸, the overlap function *w_n_* for the lenses OK2 and OK3 can be non-zero. Therefore, this section is divided into two subsections: the first subsection compares the focusing performance of four types of lenses, OC, OK1, OK2 and OK3, and the second subsection compares the two lenses OK2 and OK3, where *w_n_* is non-zero.

### 3.1. Simulation 1

In this section, the BPM is employed to simulate four types of lenses: OC, OK1, OK2 and OK3. All these lenses are ideal lenses fabricated from SU8 (Khan Malek, 2002 ▸) material with an aperture *A* = 200 µm (the medium thickness *d*_*n*_ = 0 µm) and the initial single lens boundaries are designed with the same curvature. For systematic comparison, all lenses were fabricated using the same SU8 photoresist material, maintained identical initial apertures and achieved comparable working distances. Detailed structural parameters are summarized in Table 1 ▸.

**Figure 4:**
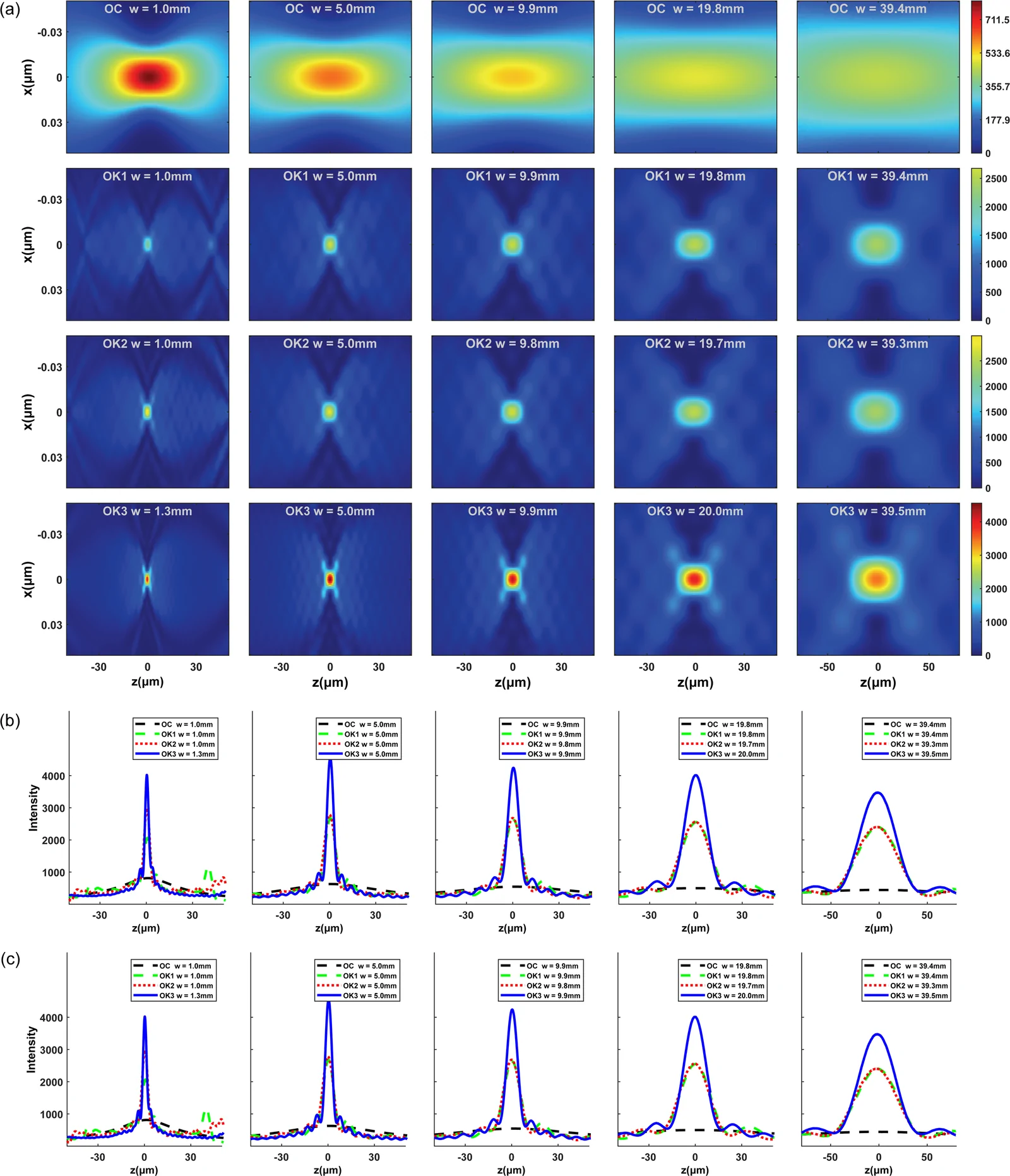
Simulation results of the BPM, showing focal spot intensity distribution and profile distribution, where *w* represents the working distance. (*a*) Comparison of optical intensities on the focal plane for OK1, OK2 and OK3. (*b*) Focal spot profiles near the focal point for OK1, OK2 and OK3. (*c*) Depths of focus near the focal point for OK1, OK2 and OK3.

To simplify the simulation setup, a plane wave with a unit intensity of 1 and an energy of 10 keV was used as the initial light source. For the *x*-direction lens unit configuration, the lateral sampling interval δ_*x*_ was set to 1 nm, while the axial propagation step δ_*z*_ along the optical axis was maintained at 0.1 µm to ensure numerical convergence. The resulting focal spot profile and intensity distribution (Fig. 4 ▸) demonstrate that the simulated focal lengths of these four lenses closely match the designed values, with all exhibiting a short depth of focus (DOF), consistent with the aberration-free focusing characteristics inherent in oval surface profiles. However, the OK3 lens design presented in this work employs a single-lens dual-focus kinoform configuration, which achieves two critical improvements in focusing compared with the OC, OK1 and OK2 lenses. The focal spot gain and FWHM results for the four lens configurations (OK1, OK2, OK3 and OC) are summarized in Tables 2 ▸ and 3 ▸.

Fig. 4 ▸ demonstrates the focusing characteristics of the four lenses (OC, OK1, OK2 and OK3). Fig. 4 ▸(*a*) exhibits the focused spot patterns, Fig. 4 ▸(*b*) compares the FWHM of these lenses under identical working distances and Fig. 4 ▸(*c*) contrasts their DOF. As revealed in Fig. 4 ▸(*b*), the OK3 lens achieves narrower FWHM values than the other three counter­parts at working distances of 1.0, 5.0, 9.9, 19.8 and 39.4 µm. Concurrently, Fig. 4 ▸(*c*) demonstrates a reduced DOF for OK3 compared with the other lenses. The superior focusing performance of OK3 over OK1 and OK2 stems from its diffraction-optimized design, which strategically minimizes the material density while preserving an optical efficiency approach derived from rigorous diffraction theory principles.

**Figure 5:**
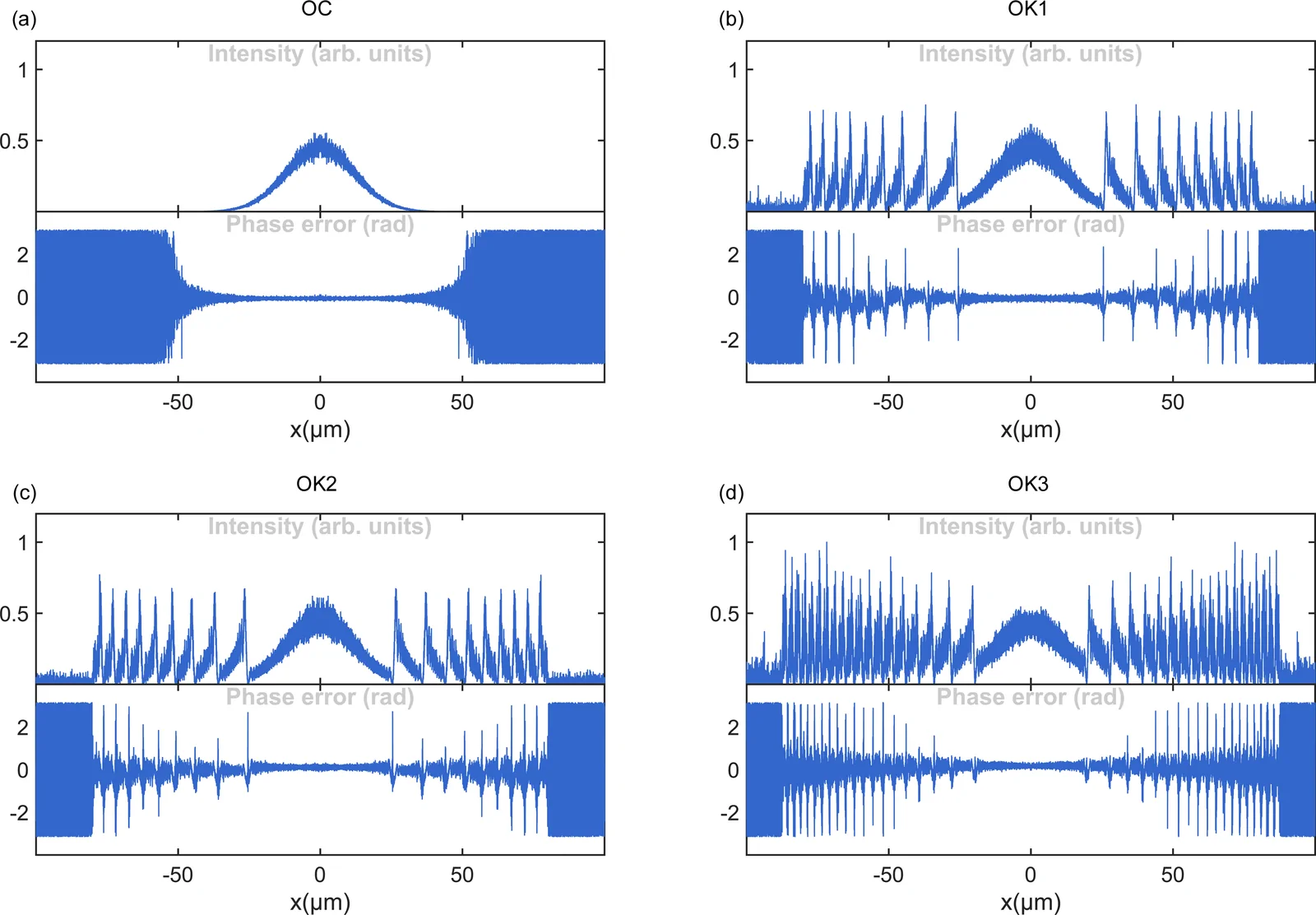
Comparative analysis of gain and phase error for 200 µm aperture lenses (OC, OK1, OK2 and OK3) with 39.4 mm working distance. The plots show the exit wavefront gain and phase error of (*a*) the OC lens, (*b*) the OK1 lens, (*c*) the OK2 lens and (*d*) the OK3 lens.

For the OC, OK1, OK2 and OK3 lenses with a working distance of 39.4 mm, the BPM was employed to compare the simulated wavefronts with their ideal counterparts. As demonstrated in Fig. 5 ▸, the relative phase differences (weighted by gain) for the four lenses OC, OK1, OK2 and OK3 are 0.0731 rad, 0.2104 rad, 0.2011 rad and 0.2637 rad, respectively, with corresponding operational efficiencies of 18.6%, 45.3%, 44.89% and 59.21%. Among them, the OK3 lens demonstrates the highest operational efficiency while also exhibiting marginally superior focusing capability compared with the other three lenses. Most importantly, the total length of the lens exhibits a significant shortening trend compared with the other three types of lenses (see Table 1 ▸).

**Figure 6:**
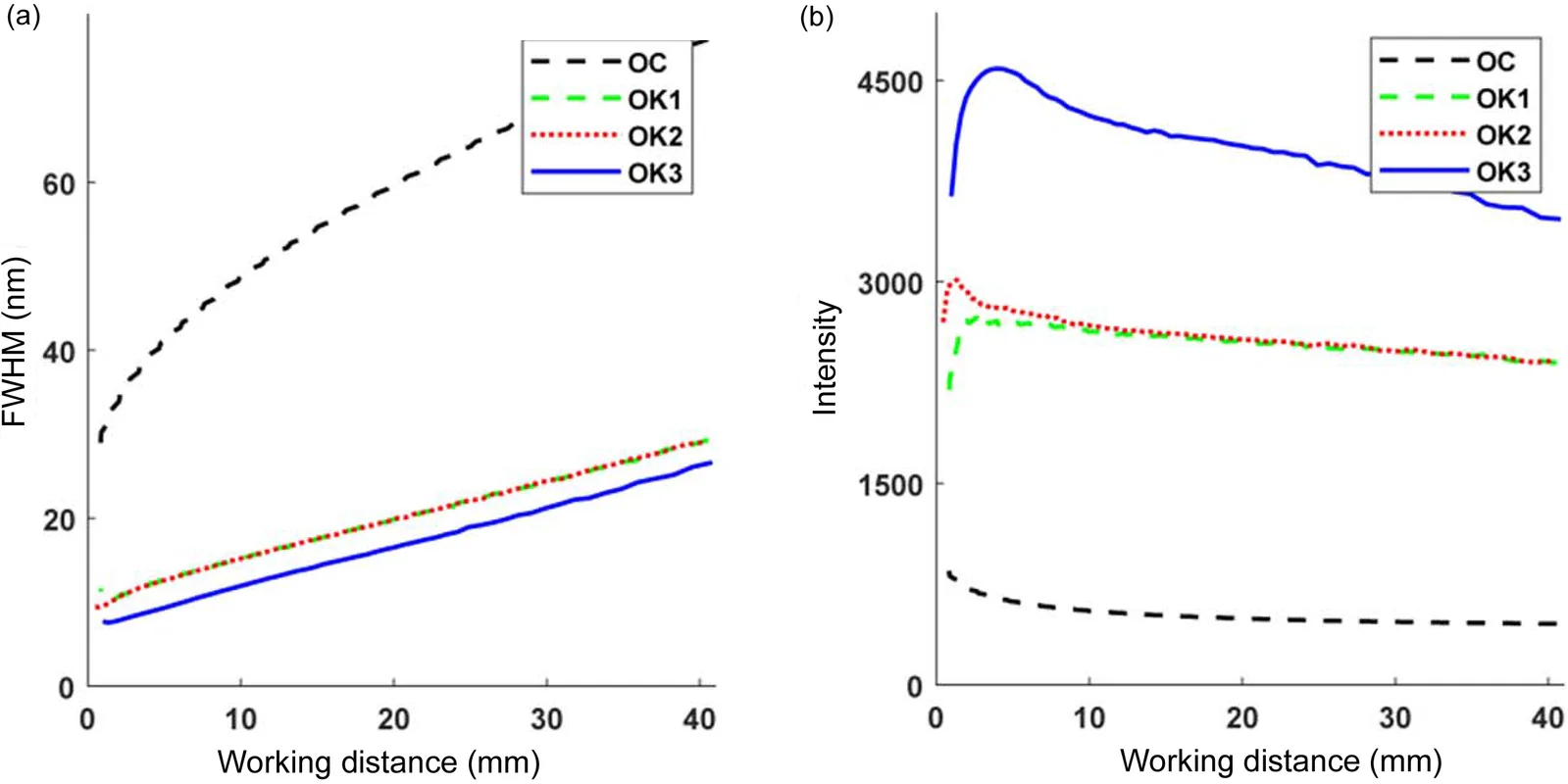
Focusing performance versus working distance for 200 µm aperture OC, OK1, OK2 and OK3 lenses. (*a*) FWHM variations and (*b*) gain variations.

Additionally, the BPM was employed to simulate four types of lenses across a working distance range of 1–40 mm, yielding variations in light intensity and FWHM with working distance as illustrated in Fig. 6 ▸. For these working distances, OC yields the largest FWHM spot sizes and the lowest intensity at the focus. OK1 and OK2 perform better than OC and comparably with each other in both respects. OK3 clearly yields the narrowest FWHM spot size and the highest intensity at all working distances. Therefore, OK3 is shown to be the best design. One might also say that OK3 achieves the same FWHM focal spot size at larger working distances than the other three choices, especially OC. Lenses with longer working distances require fewer refracting surfaces and thus are less demanding to manufacture.

### 3.2. Simulation 2

**Figure 7:**
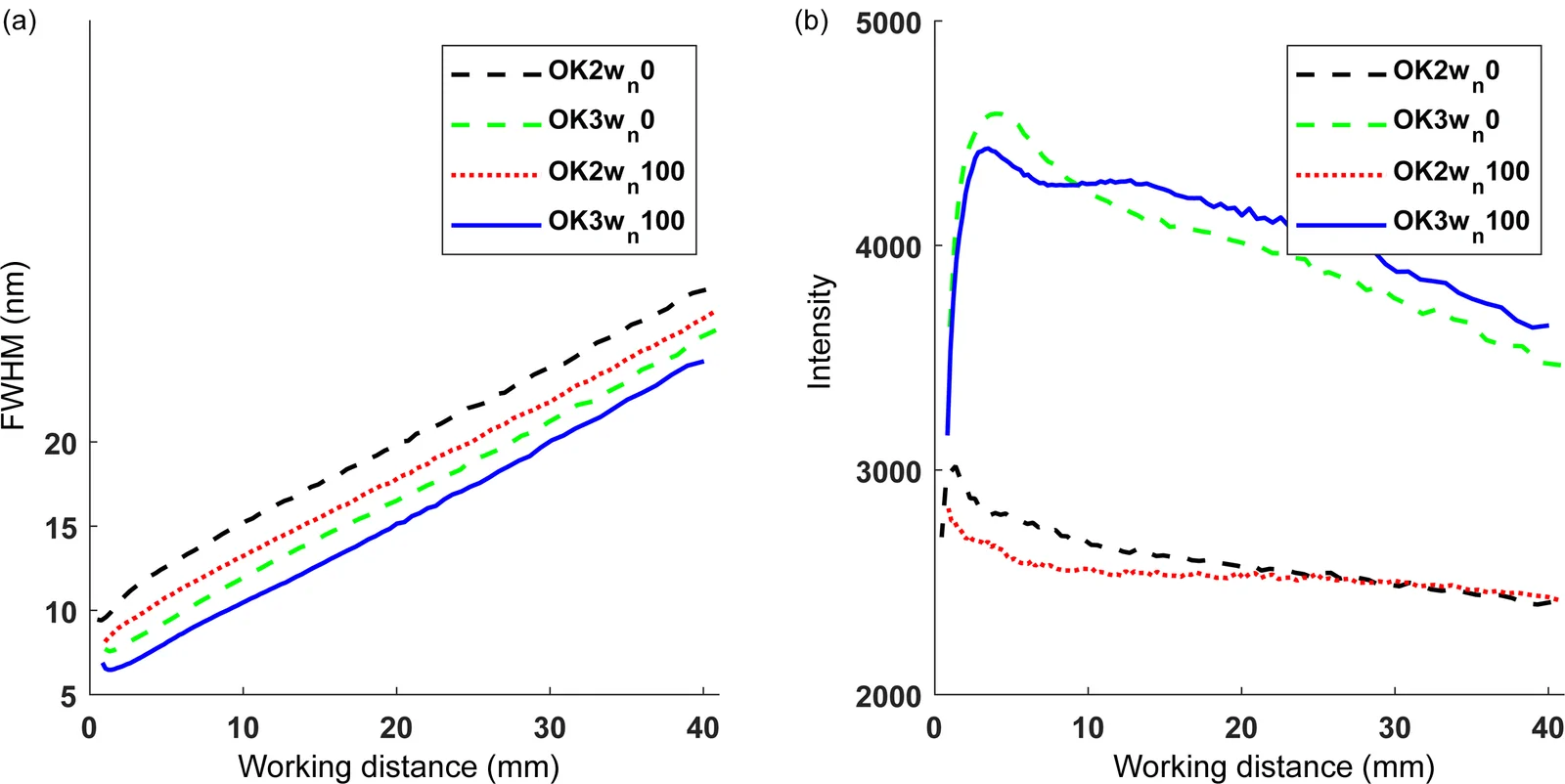
Focusing performance versus working distance for 200 µm aperture OK2 *w_n_* = 0 µm, OK3 *w_n_* = 0 µm, OK2 *w_n_* = 100 µm and OK3 *w_n_* = 100 µm lenses. (*a*) FWHM variations and (*b*) gain variations.

In this section, the BPM was employed to simulate lenses OK2 and OK3, for which the overlap function is non-zero. The initial aperture diameter of the lenses was set to 200 µm and the medium material used was SU8. The relationship between focusing performance and working distance is illustrated in Fig. 7 ▸. Fig. 7 ▸(*a*) depicts the variation in FWHM with respect to working distance, while Fig. 7 ▸(*b*) presents the gain as a function of working distance. The black dashed lines represent OK2 with an overlap function *w*_*n*_ = 0 µm, the green dashed lines represent OK3 with *w*_*n*_ = 0 µm, the red dotted lines correspond to OK2 with *w*_*n*_ = 100 µm and the blue solid lines indicate OK3 with *w*_*n*_ = 100 µm.

As illustrated in Fig. 7 ▸(*a*), for the OK2 lens, the FWHM when *w*_*n*_ = 100 µm is smaller than that when *w*_*n*_ = 0 µm, but still larger than that of the OK3 lens. For the OK3 lens, the FWHM when *w*_*n*_ = 100 µm is smaller than the case when *w*_*n*_ = 0 µm, although the extrema occur earlier than in the *w*_*n*_ = 0 µm scenario. Fig. 7 ▸(*b*) reveals that, for the OK2 lens, at larger working distances, the gain for *w*_*n*_ = 100 µm exceeds that for *w*_*n*_ = 0 µm; however, at shorter working distances, the gain for *w*_*n*_ = 100 µm is lower than that for *w*_*n*_ = 0 µm. The working distance corresponding to the maximum gain for *w*_*n*_ = 100 µm is smaller than that corresponding to the maximum gain for *w*_*n*_ = 0 µm; the same trend holds for the OK3 lens. The underlying reason for these results is that, as the number of lenses increases, the reduction in working distance is initially governed by the refractive power of the earlier lenses and subsequently influenced by the lengths of the later lenses.

## 4. Summary and prospect

In this paper, a novel kinoform lens (OK3) is proposed based on diffraction theory and the aberration-free surface profile of an oval lens. The BPM is employed to compare the performance of the 200 µm aperture OK3 lens with that of the OC, OK1 and OK2 lenses. The results indicate that OK3 exhibits superior focusing capabilities. Additionally, since OK2 and OK3 can be overlapped to reduce the lens length, we further compare the focusing performance of the OK2 and OK3 lenses with overlapping parameters *w*_*n*_ = 0 µm and *w*_*n*_ = 100 µm. It is observed that the OK3 lens with *w*_*n*_ = 100 µm achieves a smaller FWHM than that of the OK3 lens with *w*_*n*_ = 0 µm. However, with the increase in the number of lenses, the absorption also increases, resulting in reduced gain for the OK3 lens with *w*_*n*_ = 100 µm compared with that with *w*_*n*_ = 0 µm at shorter working distances. The proposed OK3 lens demonstrates improved focusing performance compared with traditional kinoform lenses and features a shorter length than conventional refractive focusing lenses, making it more suitable for the focusing of high-energy X-rays.

## Figures and Tables

**Table 1:** Key parameters of OC, OK1, OK2 and OK3

Parameter	OC/OK1	OK2	OK3
Material	SU8	SU8	SU8
Entrance aperture (µm)	200.0	200.0	200.0
Length (mm)	19.5, 27.2, 32.7, 36.3, 39.3	19.5, 27.2, 32.7, 36.3, 39.3	10.9, 16.5, 21.3, 24.4, 27.2
Working distance (mm)	39.4, 19.8, 9.9, 5.0, 1.0	39.3, 19.7, 9.8, 5.0, 1.0	39.5, 20.0, 9.9, 5.0, 1.3

**Table 2:** Focusing FWHM of OC, OK1, OK2 and OK3 lenses at identical working distances

Working distance (mm)	OC (nm)	OK1 (nm)	OK2 (nm)	OK3 (nm)
1.0	30.2	10.9	9.6	7.7
5.0	40.9	12.6	12.6	9.4
9.9	48.7	15.1	15.1	11.9
19.8	59.7	19.8	19.7	16.5
39.4	76.4	28.7	28.8	26.1

**Table 3:** Focusing gain of OC, OK1, OK2 and OK3 lenses at identical working distances

Working distance (mm)	OC	OK1	OK2	OK3
1.0	810.7	2275.0	2992.7	3637.2
5.0	623.8	2676.3	2784.5	4557.9
9.9	551.1	2642.6	2686.1	4240.2
19.8	494.8	2555.5	2573.7	4012.9
39.4	456.1	2413.3	2401.2	3478.1
